# Animal Coronaviruses Induced Apoptosis

**DOI:** 10.3390/life11030185

**Published:** 2021-02-26

**Authors:** Katerina Gioti, Christine Kottaridi, Chrysa Voyiatzaki, Dimitrios Chaniotis, Theodoros Rampias, Apostolos Beloukas

**Affiliations:** 1 Department of Biomedical Sciences, University of West Attica, 12243 Athens, Greece; agioti@uniwa.gr (K.G.); ckottaridi@bio.auth.gr (C.K.); cvoyiatz@uniwa.gr (C.V.); dchaniotis@uniwa.gr (D.C.); 2 Department of Genetics, Development and Molecular Biology, School of Biology, Aristotle University of Thessaloniki, 54124 Thessaloniki, Greece; 3 Biomedical Research Foundation of the Academy of Athens, Basic Research Center, 11527 Athens, Greece; 4 Institute of Infection & Global Health, University of Liverpool, Liverpool L69 7BE, UK

**Keywords:** animal coronaviruses, apoptosis, apoptotic pathways

## Abstract

Apoptosis is a form of programmed death that has also been observed in cells infected by several viruses. It is considered one of the most critical innate immune mechanisms that limits pathogen proliferation and propagation before the initiation of the adaptive immune response. Recent studies investigating the cellular responses to SARS-CoV and SARS-CoV-2 infection have revealed that coronaviruses can alter cellular homeostasis and promote cell death, providing evidence that the modulation of apoptotic pathways is important for viral replication and propagation. Despite the genetic diversity among different coronavirus clades and the infection of different cell types and several hosts, research studies in animal coronaviruses indicate that apoptosis in host cells is induced by common molecular mechanisms and apoptotic pathways. We summarize and critically review current knowledge on the molecular aspects of cell-death regulation during animal coronaviruses infection and the viral–host interactions to this process. Future research is expected to lead to a better understanding of the regulation of cell death during coronavirus infection. Moreover, investigating the role of viral proteins in this process will help us to identify novel antiviral targets related to apoptotic signaling pathways.

## 1. Introduction

### 1.1. Coronaviruses (CoVs)

Coronaviruses (CoVs) are the largest RNA viruses identified to date. Their genome is a positive-sense, single-stranded RNA genome (+ssRNA) that is 27−30 kb in length, which typically encodes four structural proteins, namely the spike (S), nucleocapsid (N), membrane (M), and envelope (E). Coronaviruses genome also encodes several nonstructural proteins by subgenomic mRNAs and two large polyproteins by mRNA 1. The two polyproteins are processed by viral proteases to generate more than 10 mature cleavage products [1]. Since 1937, when the first CoV was firstly isolated from an outbreak in chicken flocks (Avian Infectious Bronchitis Virus), related CoVs have been discovered in a wide range of avian and mammalian hosts, including rodents, domestic animals, and humans [2]. Coronaviruses cause a wide spectrum of diseases in humans and animals but mainly infect the respiratory and gastrointestinal mucosa [3].

**Figure 1:**
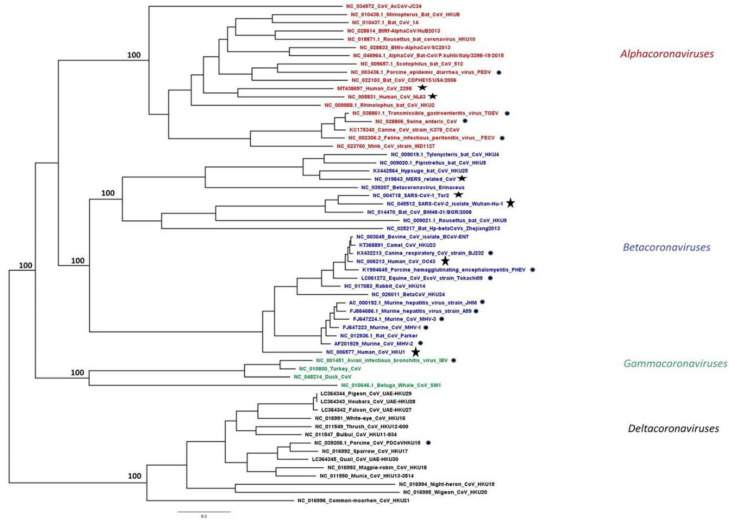
Phylogeny of human and animal CoVs. Maximum likelihood (ML) phylogeny was inferred by RAxML MPI v8.2.12 using all available complete reference genomes in the NCBI Virus database aligned using MAFFT v.7, as previously described [5]. The *alpha*-, *beta*-, *gamma*- and *deltacoronavirus* genera are coloured in red, blue, green, and black, respectively. Taxa highlighted with a star represent hCoVs, while the ones with a circle represent animal CoVs discussed in this review.

CoVs are members of the subfamily *Coronavirinae* in the family *Coronaviridae*, order *Nidovirales*. According to the latest report of the International Committee on Taxonomy of Viruses [4], CoVs are classified into four genera *Alphacoronavirus*, *Betacoronavirus*, *Gammacoronavirus*, and *Deltacoronavirus* (Figure 1). The first two genera include only mammalian CoVs, with human CoVs also clustered in each of these groups.

Viruses interfere with host factors in all steps of the viral replication cycle. Hence, host gene expression and anti-viral defense mechanisms are modulated by viruses to optimize their own replication. Two aspects of such regulations concern the progression of host cell cycle and apoptosis. Apoptosis constitutes part of the host cell defense mechanisms against viral infection [6]. Viral infection could activate a variety of signal transduction apoptotic pathways that lead to the death of the viral infected cells. Apoptosis can also facilitate the release of progeny virus and thus plays an important role in the viral life cycle during a lytic infection [7]. Intracellular pathways leading to apoptotic death can be activated either directly by viruses or indirectly by triggering cellular pathways that activate apoptotic pathways of the host cell. In any case, virus-induced apoptosis could be detrimental for the host and it is a common event in lytic viral infections. Several animal and human coronaviruses produce acute lytic infections, and apoptotic cell death can be one plausible mechanism of induced cell damage.

### 1.2. Host Cell Defense upon Infection: The Combined Effect of Antiviral Transcription and Programmed Cell Death in Establishment of Prolonged Immunity

The antiviral defense in multicellular hosts is designed to recognize and eliminate virions and virus-infected cells. In this context, after virus infection, an antiviral transcriptional program that promotes the expression of interferons (IFNs), cytokines, chemokines, and the activation of programmed cell death is initiated [8]. The transcriptional upregulation of IFN-stimulated genes limits virus replication in infected cells and induces an anti-viral state in adjacent uninfected cells [9]. Moreover, cell death via apoptosis prevents virus replication by eliminating the infected cell and additionally promotes the release of cytokines and inflammatory mediators that contribute to the chemotaxis of immune cells to the site of infection and promote prolonged immunity [10,11].

#### 1.2.1. Mechanisms of Apoptosis after Viral Infection

Innate immunity response is first initiated when the conserved structures of pathogens, referred as pathogen-associated molecular patterns (PAMPs), are detected by pattern recognition receptors (PRRs), including Toll-like receptors (TLRs) and C-type lectin receptors (CLRs), which are located on the cell surface. Upon PAMP recognition, these receptors dimerize, associate with adaptor proteins, and initiate signaling cascades that result in transcriptional upregulation of interferons (IFNs) [12].

A first-line antiviral defense also includes proteins that recognize viral DNA and RNA. Following attachment, internalized virus particles deliver their genomic material and lunch their replication program. Host proteins that function as sensors of viral DNA/RNA genomes (cGAS, ZBP1, IFI16, TLR3, RIG-I, MDA5) initiate a signaling cascade via their adaptor proteins that activate transcription factors NFĸB and IFN regulatory factor 3 (IRF3). As a result, an antiviral transcriptional program that includes upregulation of type I IFNs is initiated [13].

The binding of IFN to IFN-α/β receptor (IFNAR), leads to signaling via the Janus kinase/signal transducers and activators of transcription (JAK/STAT) pathway and leads to the transcriptional upregulation of a variety of target genes referred to as IFN-stimulated genes (ISGs) [14]. Protein kinase RNA-activated (PKR) is a well characterized IFN-stimulated gene that phosphorylates and inactivates the key translation factor eukaryotic translation initiation factor 2A (eIF2A) [15]. In this context, PKR, has been demonstrated to be a major inducer of cell death upon infection by RNA viruses [16,17].

Similarly, 2′,5′-oligoadenylate synthetase (OAS) and RNase L as IFN-stimulated genes collaborate to induce apoptosis upon virus infection. More specifically, OAS detects dsRNA in virus-infected cells and generates 2–5-linked oligoadenylates that activate RNase L to cleave host and viral RNA [18]. Thus, besides PKR, the activity of OAS–RNase L can also drive apoptosis through translational shutoff.

#### 1.2.2. Components of Apoptotic Process

Apoptotic mitochondrial events are primarily regulated through the activation of pro-survival and pro-apoptotic proteins. The Bcl-2 (B cell lymphoma-2) family of proteins constitutes a critical control point in the regulation of apoptosis. They comprise three major protein subgroups: the BH3 (Bcl-2 homology)-only proteins (e.g., Bid, Bad), Bax-like proteins (e.g., Bax, Bak) and the Bcl-2-like factors (e.g., Mcl-1, Bcl- XL) [7]. BH3-only and Bax-like proteins are essential initiators of apoptosis, while the Bcl-2-like proteins are pro-survival factors that safeguard the cells against apoptosis. An increase in the expression of pro-apoptotic proteins of the Bcl-2 family such as Bax and Bak, which can form pores in the outer mitochondrial membrane, induces the efflux of cytochrome-c from the mitochondria into the cytosol. Cytochrome-c then complexes with Apaf-1 and pro-caspase 9 to activate caspase 9, which leads to the subsequent activation of caspase-3 and -7 [8]. Bak and Bax can also be recruited to the endoplasmic reticulum (ER) and initiate apoptosis in response to cellular stress. On the other hand, Bak heterodimerization with Bcl-2-like anti-apoptotic factors, such as Mcl-1 and Bcl-XL, suppresses Bak homo-oligomerization and pore formation in unstressed, healthy cells [9].

Mitogen-activated protein kinases (MAPKs) are conserved kinases regulating critical signaling pathways including apoptosis. Four subgroups of MAPKs are identified, namely extracellular regulated kinase 1/2 (ERK1/2), ERK5, p38, and c-Jun N-terminal kinases (JNK). Among them, ERK1/2 is activated by growth factors and mitogens, whereas p38 and JNK respond to cellular stresses and/or environmental stimuli [10]. MAPKs are activated by kinase cascades. In particular, JNK is activated by MAPK kinases 4 (MKK4) or MKK7, which are phosphorylated by upstream MAPK kinase kinases. Active JNK phosphorylates c-Jun and other substrates to modulate their activities [11]. JNK pathway modulates apoptosis either by transactivation of pro-apoptotic genes or by interactions with B-cell lymphoma 2 (Bcl2) family proteins [12]. JNK-dependent activation of AP-1 upregulates expression of pro-apoptotic genes, such as Bcl2 homologous antagonist killer, Fas ligand, and tumor necrosis factor-alpha. Some transcription factors, such as p53 and p73, are also activated by JNK and promote cell death [13].

### 1.3. Viral Modulation of Apoptosis

Through the co-evolution with host cells, viruses have developed diverse strategies to overcome these anti-viral responses, evading or delaying early apoptosis long enough to generate a sufficient yield of progeny virus. In this context, many viruses encode homologs of anti-apoptotic proteins in the host cell that suppress or delay apoptosis. For instance, Epstein–Barr virus (EBV), Kaposi’s sarcoma associated γ-herpesvirus (KSHV), and mouse γ-herpesvirus (MHV) express Bcl-2 orthologs that inhibit apoptosis [19]. Other viral proteins can suppress the transcription of apoptotic proteins or suppress their activity by post-translational modifications. For instance, Tax protein, expressed by the human T cell leukemia virus type 1 has been shown to repress Bax transcription [20] while, stimulation of phosphorylation of apoptotic Bad protein by virus protein Nef (expressed by human immunodeficiency virus 1, HIV-1) and U(S)3 (expressed by herpes simplex virus 1, HSV-1) has been demonstrated to suppress activation of apoptosis [21,22]. Another strategy to suppress apoptosis, includes the inhibition of TNF receptor (TNFR) family members Fas, TNFR1, and TRAILR2. Several poxviruses such as the Shope fibroma virus express TNFR orthologs that represent soluble decoy receptors inhibiting TNF ligand-receptor interactions [23].

Conversely, specific viruses can hijack the originally protective apoptotic signaling of the host and stimulate apoptosis at later stages of infection in order to favor viral dissemination by cell breakdown. For instance, caspace -3 activation and apoptosis is essential for efficient influenza virus propagation [24]. In many cases, a direct link between the apoptotic process and the function of specific viral proteins has been established. The adenovirus E3 protein is essential for virus release and enhances virus growth in cultured cells by promoting cell death [25]. In a similar way, NS3 protein of hepatitis C virus has been demonstrated as an inducer of caspase-8 mediated apoptosis [26].

The ongoing coronavirus disease 2019 (COVID-19), caused by a new human coronavirus (hCoVs), SARS-CoV-2, highlights the importance of coronaviruses as human and animal pathogens. The cellular mechanisms exploited by coronaviruses for their optimal replication and enhanced pathogenicity warrants further investigation to deepen our understanding and, in this review, we aimed to summarize and critically review all available data on how animal CoVs induce apoptotic pathways in their host cells.

## 2. Animal Coronaviruses (CoVs) and Apoptosis

### 2.1. Alphacoronaviruses

#### 2.1.1. Porcine Epidemic Diarrhea Virus (PEDV)

Porcine Epidemic Diarrhea Virus (PEDV) is an enveloped single-stranded and positive-sense RNA virus, which belongs to the genus *Alphacoronavirus* (Figure 1). PEDV is the etiological agent for Porcine Epidemic Diarrhea (PED), an acute and highly contagious enteric disease characterized by severe watery diarrhea, dehydration, and anorexia in piglets, was first isolated and recognized in Europe in the 1970s [27]. Since then, there have been severe outbreaks of diarrheal disease in Asia and in North America posing a serious threat to the swine industry worldwide [28].

The PEDV genome is composed of a 5′ untranslated region (5′UTR) and at least seven open reading frames (ORFs): ORF 1a/1b, spike (S), ORF3, envelope (E), membrane (M) and nucleocapsid (N), and a 3′UTR in order [29].

**Figure 2:**
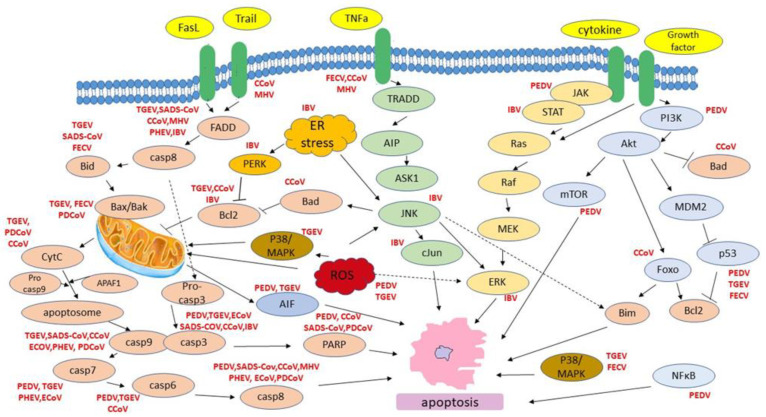
Host cell apoptotic pathways induced by animal CoVs.

Consistent with pathological changes in vivo, PEDV infection can induce morphological and biochemical changes in some cell lines in vitro, interfering with the apoptotic machinery of host cells. In particular, Kim and Lee first reported that PEDV (SM98-1 strain) induces apoptosis in Vero cells interfering with the caspase-independent mitochondrial AIF-mediated pathway in order to facilitate its replication and pathogenesis. The same results were also observed in tissues specimens collected from the duodenum, jejunum, and ileum of infected pigs [30]. The induction of apoptosis from PEDV in Vero cells was further supported by proteomic analysis, which revealed changes in the expression levels of at least 14 proteins participating in the host cells’ apoptotic pathways [31]. Besides, the induction of apoptosis as a fundamental aspect of viral replication has also been demonstrated by Oh et al. since N protein cleavage during viral replication is dependent on caspase 6 or 7 of host cells (Vero) [32]. In 2018, Chen et al. reported that different PEDV strains (CV777, SM98) could infect various host cells, including Vero, Vero-E6, and Marc-145, and cause obvious apoptotic effects such as roundup, cell fusion, cell membrane vacuolation, and syncytium formation. PEDV S1 protein was proposed to be the inducer of apoptosis, activating caspase-3 and caspase-8, which leads to AIFM1 and PARP cleavage [33]. More recently, PEDV infection of Vero cells was shown to induce p53 signaling activation and translocation to the nucleus, and p53 activation is partly mediated by ROS accumulation. These events trigger both the intrinsic and the extrinsic apoptotic pathways of infected cells [34]. On the contrary, a recently published study proposes that PEDV ORF3 protein promotes virus proliferation in Murine L (LR7) and Vero CCL-81 cells by inhibiting cell apoptosis caused by virus infection through the inhibition of caspase-3 cleavage [35]. Apart from the induction of apoptosis, PDEV also leads to G0/G1 cell cycle arrest of Vero cells though p53-dependent pathway in favor of viral replication (Figure 2) [36].

Most research on PEDV has been conducted using Vero and Marc-145 cell lines, which are derived from cells originally isolated from monkey kidneys. These cell lines greatly differ from the natural host of PEDV replication, porcine intestinal epithelial cells. Studies using the relatively new cell line IPEC-J2 (Intestinal Porcine Epithelial Cell line-J2) have also revealed the induction of apoptosis upon PEDV infection. A study in pig small intestinal epithelial cells highlighted changes in apoptosis due to PDEV (CV777 strain) infection [37]. Proteomic analysis of IPEC-J2 infected cells with different strains of PEDV supported that viral infection disrupts the apoptotic potential of infected cells by down-regulation of PI3K-AKT/mTOR signaling pathways and up-regulation of JAK-STAT and NF-κB pathways [38]. In IPEC-J2 cells, the induction of apoptosis is also accompanied with S cycle arrest upon PEDV (CV777 strain) infection. PEDV can negatively regulate the PI3K/Akt signaling pathway as most of the positive regulators (EPHA2, ITGB4, EFNA1, IL2RG, TNC, CSF3, and MYC) of the pathway were down-regulated whereas the negative regulator PTEN was up-regulated. On the other hand, data concerning the mTOR pathway were ambiguous. Some of the mTOR upstream regulators (ATP6V1G1, FZD2, and LAMTOR2) were significantly up-regulated whereas others were down-regulated (SLC3A2, SLC7A5, ITGA9, and LRP5), yet the negative regulator, PTEN, was up-regulated [39]. Nevertheless, PDEV infection interferes with multiple apoptotic pathways leading to death of the infected cells.

#### 2.1.2. Transmissible Gastroenteritis Virus (TGEV)

Transmissible gastroenteritis virus (TGEV) is an enveloped virus that contains a large, positive-sense, single-stranded, capped, and polyadenylated RNA genome of 28.6 kb [40]. The 5′-two-thirds of the TGEV genome encodes the replicase-transcription complex, Rep1a and Rep1b. During TGEV replication cycle, a 3′-coterminal nested set of subgenomic mRNAs is produced which encode other viral proteins. TGEV has four major structural proteins: the spike (S), the integral membrane protein (M), the nucleocapsid protein (N) and a small envelope protein (sM). TGEV N protein, a multifunctional phosphoprotein, plays a primary structural role in packaging the RNA genome into a helical ribonucleoprotein, as well as regulatory roles in viral RNA synthesis (replication and transcription), translation, and modulation of host cell metabolism [41]. TGEV replicates in enterocytes and provokes villous atrophy, resulting in lethal watery diarrhea and dehydration in piglets, which is considered to be a central event in the pathogenesis of TGEV infection [42].

In consistent with in vivo pathologic changes, TGEV also induces cytopathic effects (CPE) when propagated in vitro cultured cells. Initially, evidence of apoptosis caused by TGEV was shown in infected swine testicular cells, in vitro [43]. The induction of apoptosis was also observed and thoroughly characterized in PK-15 (porcine kidney cells) by Ding et al. TGEV infection promotes the activation of p38 MAPK and p53 signaling. In particular, TGEV infection decreases p300/CBP, downregulates MDM2, and promotes p53 phosphorylation at serines 15, 20, and 46, resulting in the accumulation and activation of p53 in PK-15 cells. TGEV infection also results in the transient activation of p38 MAPK in the early phase of inoculation and constant activation in the later phase of infection [44]. Evidence of ROS accumulation has also been observed which promotes the activation of p38 MAPK and p53, and subsequently p53 partly regulates ROS production [45]. p53 and ROS also provoke PARP-1 activation and cleavage which in turn mediate the AIF (apoptosis-inducing factor) translocation to the nucleus, further promoting the induced apoptotic machinery [46]. Furthermore, TGEV-induced apoptosis is dependent on viral replication in PK-15 cells and occurs through activation of FasL- and mitochondria-mediated apoptotic pathways. Hence, TGEV infection is found to up-regulate FasL and activate FasL-mediated apoptotic pathway, leading to activation of caspase-8 and cleavage of Bid. Down-regulation of Bcl-2 in combination with up-regulated Bax expression is also observed, promoting translocation of Bax to mitochondria, activation of mitochondria-mediated apoptotic pathway and the subsequent release of cytochrome c and activation of caspase-9. Both extrinsic and intrinsic activated pathways have as downstream effector caspase-3 resulting in cell apoptosis (Figure 2) [47].

Further studies on viral proteins have proposed that S1 protein and nucleocapsid (N) protein have a general function in inducing cell apoptosis in PK-15 cells. TGEV N protein suppresses cell proliferation by causing S and G2/M cell cycle arrest and apoptosis through accumulation of p53 and p21 and translocation of Bax to mitochondria in parallel with suppression of cyclin B1, cdc2, and cdk2 expression [48]. The apoptosis inducing effect of N protein has also been demonstrated in human rectal tumor cell line HRT18 by Elouet et al. TGEV infection of HRT18 cells results in the activation of caspase-3, -6, -7, -8, and -9 and cleavage of the caspase substrates eIF4GI, gelsolin, and α-fodrin. Interestingly, the TGEV nucleoprotein (N) undergoes proteolysis in parallel with the activation of caspases within the host cells [49].

Finally, microRNAs (miRNAs) of host cells play a key role in the regulation of virus-induced apoptosis. During the process of apoptosis induced by TGEV infection in PK-15 cells, the miR-27b is notably down-regulated. miR-27b directly binds to the 3′ UTR of RUNX1 mRNA and suppresses its expression. RUNX1 increases apoptosis through the regulation of Bax expression and the activities of caspase-3 and -9. Since miR-27b represses the mitochondrial pathway of apoptosis by targeting RUNX1, TGEV induces apoptosis via the down-regulation of miR-27b [50].

#### 2.1.3. Swine Acute Diarrhea Syndrome Coronavirus (SADS-CoV)

Swine acute diarrhea syndrome coronavirus (SADS-CoV) is a recently discovered coronavirus, with a 27,173-bp CoV genome that shares 95% sequence identity to HKU2-CoV, which causes severe and acute diarrhea and rapid weight loss in piglets less than six days old [51].

Vero E6 cells and IPI-2I cells infected with SADS-CoV were tested in order to clarify whether virus-induced apoptosis aids or worsens viral replication and pathogenicity. The results of the study indicated that caspase-dependent FasL (extrinsic)- and mitochondria (intrinsic)- mediated apoptotic pathways play a central role in SADS-CoV-induced apoptosis that facilitates viral replication. In particular, death receptor-mediated extrinsic pathway and mitochondrial-mediated intrinsic pathway are both activated. SADS-CoV infection was found to activate caspase-8, -9, and -3 and cleaved PARP. In addition, apoptosis signals from FasL are transmitted to activate caspase-8, which in turn cleaves Bid. Cleaved Bid activates caspase-9, which links the extrinsic and intrinsic pathways [52].

#### 2.1.4. Feline Coronavirus (FECV)

Feline coronavirus (FECV) is a spherical positive sense single-stranded RNA virus that is ubiquitous in wild and domestic *Felidae* family, being more than 90% prevalent in cats [53]. The FECV genome is approximately 30-kb in length with 11 open reading frames (ORFs) that encodes 25 structural, non-structural, and accessory proteins. This virus has two main prototypes; feline enteric coronavirus (FECV) that usually causes subclinical or mild diarrhea with restricted infection in lower small intestine and colon and feline infectious peritonitis virus (FIPV), which cause a systemic disease with granulomatous serositis with high amount of protein effusion (effusive FIP) or necrotizing and inflammatory lesions in variety of organs (non-effusive FIP). Both are mainly composed of nucleocapsid (N) protein, transmembrane (M) protein, and peplomer spike (S) protein [54]. It has been suggested that FIPV is a more virulent mutant form of FECV that arose from mutation in some sites like ORF 3c, spike (S) gene, and ORF 7b of FECV changing the enterocytes tropism to monocyte/macrophage cells. These mutations would shift the localized intestinal infection to the severe systemic infectious peritonitis [55].

Apoptosis has been postulated to play an important role during FIPV infection. Haagmans et al. found that FIPV infection is associated with T cell depletion by apoptosis even though the virus could not infect CD4+ and CD8+ T cells. Hence, it was speculated that the apoptosis of CD4+ and CD8+ T cells was caused by mediators from infected macrophages and/or intestinal epithelial cells [55]. Thereafter, it was proposed that natural TNF-alpha deriving from FIPV-infected macrophages induces apoptosis of a feline T-lymphocyte cell lines through p38-MAPK and caspases pathways [56]. In addition, PD-L1 which is involved in programmed cell death and the negative regulation of T cells immune response was proposed to mediate apoptosis of CD4+ and CD8+ T-cells, since it was found to be up-regulated in PBMCs and in a wide range of nonhematopoietic cells [57]. More recently, Watanabe et al. studied peritoneal cells where macrophages predominated from FIPV infected cats and their transcriptional profiling revealed that 60 genes related to apoptosis (i.e Fas, Traf 2, TNF, Bax, Bak, Bid, Bik) were up-regulated (Figure 2) [58].

Several organs, including the liver, lungs, spleen, and central nervous system are affected in cats that develop FIP, and the formation of lesions in these organs is accompanied by necrosis and pyogenic granulomatous inflammation. In vitro studies in non-haemopoetic cell line also revealed the induction of apoptosis. Transcriptional profiling of CRFK (Crandell-Rees Feline Kidney Cell cells) infected with FIPV revealed that the expression of genes from apoptosis cluster is altered and the induction of apoptosis was correlated with p53, p38 MAPK, VEGF, TNF, and chemokines/cytokines signaling pathways [59,60].

#### 2.1.5. Canine Coronavirus (CCoV)

Canine coronavirus (CCoV) is a single positive-stranded RNA CoV responsible for diarrhea, vomiting, dehydration, loss of appetite and occasional death in puppies [61]. Two serotypes of CCoVs were described, CCoV-I and -II, sharing about 90% sequence identity in most of their genome [61,62].

The CCoV 1–71 strain was firstly described by Ruggieri et al. to induce apoptosis in A-72 cell line (canine fibrosarcoma) which was caspase-3 dependent [63]. Testing in CCoV type II, which is the only CCoV that grows in cell cultures, revealed more information concerning the induction of apoptosis. CCoV-II triggers apoptosis in A-72 cells by activating initiator (caspase-8 and -9) and executioner (caspase-3 and -6) caspases. The proteolytic cleavage of poly (ADP-ribose) polymerases (PARPs) was also observed, confirming the activation of executioner caspases. Furthermore, CCoV-II infection resulted in truncated bid (tbid) translocation from the cytosolic to the mitochondrial fraction, the cytochrome c release from mitochondria, and alterations in the pro- and anti-apoptotic proteins of bcl-2 family, indicating that both intrinsic and extrinsic pathways are involved; yet apoptosis does not play a role in facilitating viral release [64]. Further studies from the same group revealed that FOXO transcription factors mediate pro-apoptotic effects of CCoV-II, in part due to activation of extrinsic apoptosis pathway, while some Sirtuin family members (such as SIRT3 and SIRT4) may be involved in intrinsic apoptotic pathway. SIRT1 is a key regulator of cell defenses and survival in response to stress [65], and deacetylates and represses FOXO1 dependent apoptosis [66]. Moreover, CCoV-II leads to a remarkable increase in the expression of TNF-related apoptosis-inducing ligand (TRAIL) in parallel with a slight up-regulation of FasL/Fas. Bax translocation into mitochondria and decreased bcl-2 expression was also observed (Table 1) [67].

### 2.2. Betacoronaviruses

#### 2.2.1. Μurine Hepatitis Virus (MHV)

Μurine hepatitis virus (MHV), is a large, enveloped, single-stranded, positive-sense RNA *Betacoronavirus*, a member of the *Coronaviridae* family (Figure 1). There are many strains of MHV that exhibit different tropisms and levels of virulence. As a natural pathogen of mice, normally infects the liver, gastrointestinal tract, and central nervous system (CNS), causing a wide range of diseases, including hepatitis, gastroenteritis, and acute and chronic encephalomyelitis [68]. The infections caused by this virus constitute models for the study of encephalitis and demyelinating diseases such as multiple sclerosis (MS), hepatitis [69,70] and severe acute respiratory syndrome [71].

There are two neurotropic strains that are commonly studied, named A59 and JHM. A59 is a tissue culture-adapted strain that infects the liver as well as the brain. A59 causes moderate to severe hepatitis and, as far as the brain is concerned, mild encephalitis and demyelination [72,73]. JHM was isolated from a paralyzed mouse [68] and subsequently serially passaged in mouse brains, after which various clones were isolated with different levels of neurovirulence. MHV-induced hepatitis has been studied using several strains, including the highly hepatovirulent MHV-3, which is the most commonly strain used to study the pathogenesis of MHV-induced hepatitis, MHV-2 and the more moderate hepatotropic A59 strain [74]. The MHV-1 strain is primarily pneumovirulent and induces pneumonitis that is highly mouse strain dependent. MHV-1 infection of A/J mice strain provides a mouse model for the pathogenesis of SARS-CoV in humans [75]. The cellular receptor for murine coronavirus mouse hepatitis virus is a member of the carcinoembryonic antigen (CEA) family of glycoproteins in the immunoglobulin superfamily [76].

The MHV virion contains a helical nucleocapsid consisting of nucleocapsid protein (N) bound to a positive sense RNA genome. The viral envelope contains spike peplomers (S), small envelope protein (E), and membrane protein (M). Depending on the viral strain, the viral envelope may also contain hemagglutinin-esterase protein (HE) and the internal protein (I) [77]. E protein appears to play a role in host virus interaction, specifically in the induction of apoptosis. E induces apoptosis in vitro in MHV-A59-infected 17Cl-1 murine cells via a caspase-dependent mechanism. The inhibition of MHV-induced apoptosis promotes virus production late in infection, suggesting that apoptosis may be a host response that limits the level of virus production [78].

Belyavskyi et al. [79] studied the possible induction of apoptosis after the infection of two different mice strains, the BALB/c and the A/J with MHV-3. As macrophages play a central role in the pathogenesis of MHV-3-induced hepatitis, they used three different methods to detect apoptosis in these cells. Apoptosis was observed in macrophage cells coming from both experimental models and the variation observed concerned the percentage of apoptotic cells when MHV-3 virus infected BALB/c and A/J were compared.

In a later study, Leibowitz and Belyavskaya [80] furtherly investigated the apoptotic effect of the macrophage infection by MHV, by utilizing caspase inhibitors. After they treated A/J mice with pan-caspase inhibitor Z-V AD-FMK and infected the mice with MHV-3, they observed an increased hepatic viral load. They proposed that the rapid development of apoptosis during MHV -3 infection prevents the expression of the fgl2 prothrombinase protein which results in fibrin deposition and hepatic necrosis. Macrophages from BTLA-deficient (B and T lymphocyte attenuator) mice rapidly lose viability following MHV-3 infection and this effect is due to rapid, TNF-related apoptosis-inducing ligand-dependent apoptosis of MHV-3-infected macrophages [81].

Two studies show apoptotic T cells, macrophages, astrocytes, and oligodendrocytes in MHV-(JHM) infection [82,83]. Three different biologic phenotypes of MHV, related to whether they cause demyelination or not, were used to examine their differential effect on the induction of apoptosis by Schwartz et al., [84]. During acute infection, apoptosis was found in the livers of all three viral phenotypes, as all three viruses cause hepatitis, while they showed that apoptosis may play an important role in both acute and chronic MHV disease, most significantly in demyelination. In vitro, cultured murine oligodendrocytes are susceptible to MHV–induced apoptosis through FAS spike glycoprotein interactions [85]. Lee et al. [86] detected apoptotic changes in the thymus of mice infected with MHV-2 by the microscopic observation of histopathological changes of the thymus.

#### 2.2.2. Porcine Hemagglutinating Encephalomyelitis Virus (PHEV)

Porcine hemagglutinating encephalomyelitis virus (PHEV) is a positive, non-segmented, single-stranded RNA coronavirus belonging to betacoronavirus genus within the coronaviridae family (Figure 1) [87]. PHEV causes vomiting and wasting disease, and encephalomyelitis in piglets under three weeks old [88]. This virus spreads to the CNS via peripheral nerves, and nerve cells are one target for viral replication [89]. The serial propagation of PHEV is feasible in several porcine cell culture systems, e.g., primary pig testis cells, secondary pig thyroid cells as well as swine kidney and swine testis cell lines. However, the mechanisms inducing death in PHEV-infected cells remains largely unknown.

Studies in apoptosis mechanisms induced by PHEV revealed that the activities of the effecter caspase, caspase-3, and the initiator caspases, caspase-8 and caspase-9, which are representative factors in the death receptor-mediated apoptotic pathway and the mitochondrial apoptotic pathway, respectively, were increased in PHEV-infected PK-15 cells [90]. In addition, changes in global gene expression in the cerebral cortex of PHEV-infected mice investigated using DNA microarray analysis revealed that apoptosis inducing proteins bak1, caspase 1,3,4,7,8,12 were up-regulated after 5 day infection [91].

#### 2.2.3. Equine Coronavirus (ECoV)

Equine coronavirus (ECoV) is a relatively newly recognized enteric virus of adult horses that has been associated with fever, lethargy and anorexia, as well as colic and diarrhea. The ECoV-NC99 genome comprises 30,992 nucleotides (nt), excluding the 3′ poly (A) tail, and has a GC content of 37.2%. Analysis of the ECoV-NC99 genome reveals 11 potential ORFs (1a, 1b, 2–8, 9a and 9b). The ORFs 1a and 1b encode the replicase polyproteins pp1a and pp1ab. The ORFs 2–8, 9a and 9b encode structural and accessory proteins NS2, HE, S, p4.7, p12.7, E, M, N, and I, respectively [92]. Sporadic cases and outbreaks have been reported with increased frequency since 2010 from Japan, the USA and more recently from Europe [93].

In in vitro studies, infected MDBK cells (bovine kidney-derived cell line) with ECoV exhibited cytopathic effects such as cell rounding, detachment and chromatin fragmentation. Caspase-3/7 activity was increased, and caspase-8 and caspase-9 activities were increased suggesting that ECoV can induce caspase-dependent apoptosis in MDBK cells via both the death receptor-mediated and mitochondrial apoptotic pathways (Figure 2) [94].

### 2.3. Gammacoronavirus

#### Avian Infectious Bronchitis Virus (IBV)

Avian infectious bronchitis virus (IBV) is a gamma coronavirus in the *Coronaviridae* family, which has been identified as the causative agent of infectious bronchitis (IB) as well as serious acute viral respiratory and urogenital diseases in commercial chicken flocks worldwide [95]. Infected chickens develop respiratory symptoms, kidney and oviduct lesions, reduced egg production with poor egg quality, and possible secondary complications [96]. IBV can replicate within the epithelial surfaces of the kidneys and cause granular degeneration, vacuolation, and desquamation of the tubular epithelium, and massive infiltration of heterophils in the interstitium. IBV-induced kidney lesions are typically characterized by interstitial nephritis and tubule lesions that are most prominent in the medulla [97].

The genome-length mRNA1 of coronavirus IBV encodes two overlapping replicase proteins in the form of polyproteins 1a and 1ab, which are processed by viral proteases into 15 nonstructural proteins (Nsp2-Nsp16) [87,98]. The subgenomic mRNAs encode four structural proteins: the highly glycosylated spike protein (S), the small membrane-associated envelope protein (E), the integral membrane protein (M) and the phosphorylated nucleocapsid protein (N), are encoded by different subgenomic mRNAs [87]. In addition, several accessory proteins, such as 3a, 3b, 5a, and 5b are also encoded by subgenomic mRNAs.

Liu et al. first reported that both necrosis and apoptosis may contribute to the death of infected Vero cells in lytic IBV infection. The induced apoptosis was caspase-dependent characterized by caspase-3 activation and poly (ADP-ribose) polymerase degradation. Among the 11 IBV-encoded proteins, 58-kDa mature cleavage product was proposed to induce apoptotic changes in cells [99]. Another study, however, revealed that IBV explores a p53-independent mechanism to induce apoptosis in cultured mammalian cells [100].

A transcriptomic analysis of IBV-infected Vero cells revealed an up-regulation at the transcriptional level of pro-apoptotic Bak [101]. In order to understand the interactions between host and virus, Cong et al. also conducted a gene expression-transcriptomic profiling of chicken kidney tissue after nephropathogenic IBV infection. Among the altered expressed proteins were positive apoptosis regulation proteins such as BCL2-antagonist/killer 1 and Fas as well with negative apoptosis regulation proteins (e.g., clusterin and microphthalmia-associated transcription factor) [102]. Another proteomic analysis of chicken kidney cells infected with the very virulent SCDY2 strain apoptosis revealed that more genes functionally related to apoptotic processes were significantly up-regulated. In particular, the apoptotic cluster contained 91 nodes associated with 1382 interactions (edges) and the core nodes were BCL2L1, GADD45, and STAT3, involved in the intrinsic and extrinsic apoptotic pathways. Moreover, the ratio of Bax/Bcl2 expression level increased after virulent IBV infection. These data suggested that the pathogenicity of virulent nephropathogenic IBV might be largely related to the ability to induce apoptosis in kidney cells. Besides, cell apoptosis promoted replication and spread of the virus, since the number of apoptotic cells in kidney post nephropathogenic IBV infection were positively related to the viral titer reached in these cells [103].

It has been well established that the endoplasmic reticulum (ER) is closely associated with coronavirus replication. The induction of the unfolded protein response (UPR) is an adaptive cellular response to endoplasmic reticulum (ER) stress that allows a cell to reestablish ER homeostasis. However, under severe and persistent ER stress, prolonged UPR may activate unique pathways that lead to cell death. IBV infection of Vero cells was shown to cause ER stress leading to induction of PERK and PKR, and subsequent phosphorylation of eIF2a. Thereafter, eIF2a induces the expression of ATF4, ATF3 and GADD153. GADD153 induces TRIB3 and exerts proapoptotic activities via suppressing Bcl2 and antagonizing the ERK survival kinases, therefore promoting apoptosis [104].

Recent studies have shed more light on induced apoptotic pathways upon IBV infection. Thus, JNK pathway is found to be activated in cells infected IBV leading to induction of apoptosis without affecting viral replication. The upstream MAPK kinases and particularly MKK7 were shown to be responsible for JNK activation which served as a pro-apoptotic protein modulating the anti-apoptotic protein Bcl2; yet the pro-apoptotic activities of JNK was mediated independent of c-Jun which probably promotes cell survival [105]. A recent study revealed that ERK1/2 kinases activate cFOS which in turn suppresses apoptosis of the infected cells at early to intermediate phases of the IBV infection cycle in favor of viral replication [106].

Specific viral proteins such as S1 and M are proposed to interfere with the apoptotic potential of host cells. Porcine epidemic diarrhea virus S1 protein is the critical inducer of apoptosis. N-linked glycosylation of M protein was shown to contribute to the induction of apoptosis during IBV infection in parallel with rendering a certain enhancement effect on the replication and pathogenesis of IBV in vivo [107].

Induction of apoptosis is also reported for immune cells. Han et al., established chicken macrophage HD11 cells infected with IBV. The induced apoptosis was dependent on activated caspase-8 by the Fas/Fas ligand (FasL)-mediated signaling pathway and activated caspase-9 by the B-cell lymphoma 2 (Bcl-2) family-mediated signaling pathway. Both activated caspases resulted in caspase-3 activation suggesting that IBV-induced apoptosis is triggered via both extrinsic and intrinsic pathways which relies on viral replication (Table 1) [108].

### 2.4. Deltacoronaviruses

#### Porcine Deltacoronavirus (PDCoV)

The PDCoV genome is approximately 25.4 kb in length and is composed of a 5′ untranslated region (UTR), at least six open reading frames (ORF1a, ORF1b, and ORF2 through 5), and a 3′ UTR. The first two large ORF1a and 1b comprising the 5′ two-thirds of the genome encode two overlapping replicase polyproteins via a −1 ribosomal frameshift. Subsequent post-translational processing of the polyproteins by viral proteases results in 15 mature nonstructural proteins (nsp2–16). The remaining ORFs in the 3′-proximal region code for the four canonical coronaviral structural proteins, spike (S), membrane (M), envelope (E), and nucleocapsid (N), as well as three accessory proteins, nonstructural gene 6 (NS6), NS7, and NS7a [109]. PDCoV causes acute diarrhea, vomiting, dehydration, and mortality in nursing pigs. The disease is clinically and pathologically similar to porcine epidemic diarrhea virus (PEDV) and transmissible gastroenteritis virus (TGEV), but with reportedly lower mortality rates [110].

Experimental infection studies showed that PDCoV infects large numbers of villous epithelial cells of the small intestine at 3–4 days after oral inoculation. Infected enterocytes appeared to acutely undergo vacuolar, or hydropic, degeneration and exfoliated extensively from the villous epithelium, followed by villous atrophy. This process appeared to be associated with necrosis of infected cells [110]. Thereafter, the induced cell death was evaluated in infected enterocytes in vivo and infected LLC porcine kidney (LLC-PK) and swine testicular (ST) cells in vitro by Jung et al. PDCoV did not induce apoptosis in the infected intestinal enterocytes in vivo, but in the two infected cell lines of swine origin, LLC-PK and ST cells [111].

Processes of cell death were firstly examined in ST cells. PDCoV infection was found to stimulate mitochondrial outer membrane permeabilization either via Bax recruitment or mitochondrial permeability transition pore opening to permit the release of apoptogenic cyt c into the cytoplasm, thereby leading to execution of the caspase-dependent intrinsic apoptosis pathway to facilitate viral replication in vitro [112].

Proteomic analysis of LLC-PK (porcine kidney) infected cells revealed that PDCoV may utilize the apoptosis pathway of host cells to achieve maximum viral replication. In particular, PDCoV infection caused the upregulation of caspase-3, caspase-7 and caspase-8 and significantly downregulated the expression of PARP1. Therefore, it was speculated that after PDCoV infection, host cells upregulate the expression of caspase-3, caspase-7, and caspase-8 to promote apoptosis. On the other hand, cleavage of PARP1 by caspase-3 may be used to downregulate the expression of PARP1 to accelerate the occurrence of apoptosis (Table 1) [113].

## 3. Discussion

Recent experimental work on the cellular responses to infection by SARS-CoV and SARS-CoV-2 have demonstrated a complex manner by which human coronaviruses hijack cellular signaling pathways and modulate apoptosis. The apoptosis of infected cells has also been extensively observed among a wide variety of coronaviruses that infect a broad range of animal species suggesting that its stimulation is a general feature of both human and animal coronaviruses that favors propagation. In animal coronaviruses, apoptosis has been mainly studied by ex vivo experiments while both apoptosis and necrosis have been also observed mainly in immune cells as well as in enteric and respiratory epithelia from infected animals.

Despite the genomic diversification of animal coronaviruses and the differences in tissue tropism within their group, two major apoptotic pathways-the extrinsic one that includes the interaction of death ligands (FasL, TNF) to respective death receptors (DRs) and the intrinsic one, that requires mitochondrial outer membrane permeabilization have been primarily associated with animal coronavirus infection (Figure 2). In animal coronaviruses that induce death receptor activation, caspase-8 was identified to play a major role in apoptosis induction. In the extrinsic pathway, activated caspase-8 cleaves Bid and thus, promotes the release of mitochondrial cytochrome c. The latter is a crucial step for apoptosome formation and caspase-9 cleavage/activation [114]. Among the death receptor family members TNF-R1, CD95 (FAS/APO-1), DR3, TRAIL-R1/DR4, TRAIL-R2/DR5, and DR69 are so far the best characterized.

On the other hand, proapoptotic BCL-2 family members BAX and BAK as well as caspase-9 were identified as essential effectors of cell death in intrinsic (mitochondrial) apoptotic pathway. Interestingly, in many members of animal coronaviruses (e.g., PDEV, TGEV, SADS-CoV, CCoV, PHEV, ECoV, IBV), observed apoptosis was associated by activation of both extrinsic and intrinsic pathways suggesting that virus infection affects a complex network of signaling pathways related to programmed cell death.

Previous studies have demonstrated that viral proteins can induce apoptosis via different ways. For example, proteinases 2A and 3C encoded by poliovirus terminate cap-dependent translation in host cells by cleavage of initiator factor eIF4G and thus induce apoptosis [115,116]. A direct interaction of NS3 protein from hepatitis C virus with caspase-8 has been demonstrated to elicit apoptosis [26] while adenovirus encoded E1A protein is known to suppresses the expression of the antiapoptotic Bcl-2 family member myeloid cell leukemia 1 (Mcl-1), promoting apoptosis [117]. Notably, PB1-F2 protein encoded by Influenza virus subtypes (H3N2, H5N1), viral protein R (Vpr) encoded by human immunodeficiency virus (HIV-1) and reovirus μ1 protein have a direct role in intrinsic apoptotic pathway, forming pores in the mitochondria and thus promoting cytochrome c release [118,119,120].

Regarding the human coronaviruses, ex-vivo experiments have demonstrated that upon SARS-CoV infection, viral 7a protein directly interacts and forms a complex with antiapoptotic Bcl-XL protein and other prosurvival factors (Bcl-2, Mcl-1, and A1), promoting apoptosis in a caspase-3 dependent manner [121]. Similarly, SARS-CoV 3C-like protease (3CLpro) has implicated in activation of caspase-3 and caspase-9 induced apoptosis in HL-CZ human promonocytic cells [122]. Other studies, in both SARS-CoV and SARS-CoV-2 have shown that viral protein ORF3a can induce a caspase dependent apoptosis in Vero E6, HEK293T, and HepG2 cells. Notably, ORF3 driven programmed cell death is mediated through the extrinsic apoptotic pathway and includes activated death receptor signaling [123,124,125].

In animal coronaviruses, the specific role of viral proteins to apoptosis induction is not adequately studied. Experimental evidence exists for Porcine epidemic diarrhea virus (PEDV) S1 spike protein that is considered as the critical inducer of apoptosis [33]. Similarly, PDEV Nsp1 has also been implicated as an essential effector of apoptosis [126]. Expanding our knowledge about the functional role of specific viral proteins in apoptosis induction among *Alphacoronavirus*, *Betacoronavirus*, *Gammacoronavirus*, and *Deltacoronavirus* will help us to better understand the aspects of pathogenesis of coronaviruses. Moreover, the molecular mechanisms of cross talk between apoptosis, viral genome replication, and viral propagation offers an opportunity for pharmaceutical targeting and development of therapeutic approaches for human and animal coronavirus infections.

## 4. Conclusions

Virus and host cell relationship is a complicated interplay and there are numerous viral and cellular factors involved in viral infection and consequential pathogenesis. As intracellular obligate parasites, viruses have evolved various strategies to hijack the cellular machinery and key cell cycle pathways. In this review, we first collate all available published data on how viral factors from numerous animal coronaviruses could manipulate the host cell to expedite its own replication cycle inducing apoptotic pathways and pathogenesis.

## Figures and Tables

**Table 1:** A synopsis of the apoptotic pathways induced by animal CoVs discussed in this review.

Coronavirus	Strain	In Vitro/In Vivo	Apoptotic Pathway	Ref.
***Alphacoronaviruses***				
**PEDV** ***Host: swine***	SM98-1	Vero Tissue specimens from duodenum, jejunum and ileum	↑AIF	[30]
	AJ1102	Vero	12 apoptotic proteins	[31]
	8aa and KD	Vero	caspase 6 or 7	[32]
	CV777 or SM98	Vero, Vero-E6, and Marc-145 cells	↑caspase-3, ↑caspase-8, AIFM1 and PARP cleavage	[33]
	Shaanxi	Vero	↑ p53, ↑ ROS	[34]
	DR13, CV777, rDR13^att^-ORF3^CV777^ rDR13^att^-ORF3^NY^ rDR13^att^-∆ORF3	LR7, Vero CCL-81	↓caspase-3 Cleavage	[35]
	CH/SXYL/2016	Vero	G0/G1 arrest, ↑p53	[36]
	CV777	Vero, immortalized pig small intestinal mucosal epithelial cells	↑apoptosis	[37]
	YC2014, CV777	IPEC-J2	↑JAK-STAT, ↑NF-kB ↓PI3K-AKT/mTOR	[38]
	CV777	IPEC-J2	↓PI3K/Akt ↑mTOR upstream regulators (ATP6V1G1, FZD2 and LAMTOR2), ↑ PTEN	[39]
**TGEV** ***Host: swine***	Shaanxi	PK-15	↓p300/CBP, ↓MDM2 ↑p53, p38 MAPK	[28]
	Shaanxi	PK-15	↑ROS, ↑p53, p38 MAPK	[45]
	Shaanxi	PK-15	↑AIFM1, PARP cleavage, ↑ROS	[46]
	Shaanxi	PK-15	↑Fas/FasL, ↑Bid, ↑Bax, ↓Bcl-2 ↑ cleaved PARP, ↑caspase 8,9,3	[47]
	Shaanxi	PK-15	S and G2/M cycle arrest ↑p53, ↑p21	[48]
	Purdue-11	HRT18	caspase 3,6,7,8,9	[49]
	Shaanxi	PK-15	miR-27b, ↓RUNX1 ↓Bax, caspase 3,9	[50]
**SADS-CoV** ***Host: swine***	SADS-CoV	Vero, IPI-2I	↑ Fas/FasL ↑ caspase 8,9, 3, PARP cleavage	[52]
**FECV** ***Host: Feline (cat)***	79-1146	Feline peripheral blood mononuclear cells (PBMC), CD4^+^ cells, CD8^+^ cells, CD21^+^ cells, peritoneal exudate cells (PEC), alveolar macrophages, and WEHI-164 murine sarcoma cells	↑TNF-α	[56]
	79–1146	CRFK	↑PDL1	[57]
	FIPV-m3c-2	Peritoneal cells	↑ Fas, TNF, Bax, Bak, Bix, Bid, Traf2	[58]
	FIPV 79-1146	CRFK	↓TNF, TGFbeta, STAT3	[59]
	FIPV 79-1146	CRFK	p53, p38 MAPK, VEGF	[60]
**CCoV** ***Host: Canine (dog)***	Type I, 1–71	A-72	↑ caspase 3	[63]
	Type II, S/378	A-72	PARP, Bid cleavage ↑ caspase 8,9,3,6	[64]
	Type II, S/378	A-72	↑TRAIL, ↑ Fas/FasL, ↓Bcl-2	[67]
***Betacoronaviruses***				
**MHV** ***Host: mouse***	A59	17Cl-1	caspase dependent	[78]
	MHV-3	A/J and BALB/c macrophages	fgl2 prothrombinase	[79]
	MHV-3	BTLA-deficient (BTLA-/-) mice	TRAIL	[81]
	A59	oligodendrocytes	apoptosis	[84]
	JHM	CG-4	↑Fas/FasL/FADD/procaspase 8	[103]
	MHV-2	thymus	apoptosis	[86]
**PHEV** ***Host: swine***	HEV-67N	PK-15	↑ Fas/FasL ↑ caspase 8,9, 3, PARP cleavage	[90]
	67Ν	Four- to six-week-old female BALB/c mice	↑bak1, ↑caspase 1,3,4,7,8,12	[91]
**ECoV** ***Host: horse***	NC99	MDBK	↑caspase 3,7,8,9	[94]
***Gammacoronaviruses***				
**IBV** ***Host: avian***	Beaudette	Vero	↑ caspase 3, ↑ cleaved PARP	[99]
	Beaudette	Vero	p53 independent	[100]
	Beaudette	Vero	↑ Bak	[101]
	ck/CH/LDL/091022	Chicken kidney tissue	↑Bcl2, Fas, ↓clusterin	[102]
	SCDY2 SCK2	Chicken kidney tissue	BCL2L1, GADD45, STAT3	[103]
	Beaudette	Vero	↑ PERK, PKR, ATF3, ATF4, GADD153, TRIB3,↓ Bcl2, ERKs	[104]
	Beaudette	Vero, H1299, Huh-7	↑ MKK7, JNK, ↓Bcl-2	[105]
	Beaudette	Vero, H1299	↑ ERK1/2, ↑cJUN, JUNB, JUND, cFOS, FOSB	[106]
	Beaudette	Vero	PARP cleavage	[107]
	Beaudette	macrophage HD11	↑ Fas/FasL,↑ caspase 8,9, 3 ↓Bcl-2	[108]
***Deltacoronaviruses***				
**PDCoV** ***Host: swine***	KNU16-07	ST cells	Bax, cytochrome C, caspase 9	[112]
	CH/XJYN/2016	LLC-PK	↑ caspase 8,7,3, ↓ PARP	[113]
